# The complete mitochondrial genome of *Lucidina vitalisi* (Coleoptera: Lampyridae) and its phylogenetic analysis

**DOI:** 10.1080/23802359.2025.2590334

**Published:** 2025-11-19

**Authors:** Xiao-Hua Guo, Xiao-Li Fan, Zi-Long Zhong, Yan-Yun Xiong, Su-Mei Wu, Jin-Yang Li, You-Jun Wu

**Affiliations:** a College of Ecology, Lishui University, Lishui, China; b Ecological Forestry Development Center of Suichang County, Lishui, China; c Qingyuan Conservation Center, Qianjiangyuan–Baishanzu National Park, Lishui, China

**Keywords:** Firefly, East Asian region, conservation genetics, mitochondrial DNA

## Abstract

We present the complete mitochondrial genome of the East Asian “dark” firefly *Lucidina vitalisi* (Coleoptera: Lampyridae: Lampyrinae). The circular mitogenome measures 14,882 bp and includes the standard set of 37 genes along with a 259-bp control region. Phylogenetic analysis based on the 13 protein-coding genes places *L. vitalisi* as sister to *Lucidina* sp., within the Lampyrinae subfamily, distinct from the Luciolinae subfamily. This mitogenome serves as a validated genomic resource for *Lucidina*, refines its phylogenetic position, and supports future comparative, population, and conservation genetic studies of East Asian fireflies.

## Introduction

*Lucidina vitalisi* (Pic 1917) is an East Asian “dark” firefly (weakly luminous or chiefly diurnal). Males bear conspicuously serrate antennae; the pronotum is typically red and often carries a median dark vitta, and the abdominal apex has a small punctiform light organ (females are slightly larger) (Jeng 2008). Dark-type fireflies generally rely on pheromones rather than bright courtship flashes and show only faint or no luminescence during the adult stage, in contrast to nocturnal flashing lineages (Fallon et al. 2022; Zurita-García et al. 2022).

Taxonomically, *Lucidina* belongs to Lampyrinae. Recent morphological and systematic studies suggest affinities with the tribe Lucidotini, although relationships within the genus and to allied lineages are still being resolved; concomitantly, corrections to the type-species designation and catalog updates have been proposed (Martin et al. 2017; 2019; Keller and Martin 2024; Viana et al. 2025). These advances have clarified tribal assignments and intergeneric relationships among East Asian “dark” fireflies, but species-level molecular evidence remains relatively scarce.

Mitochondrial genomes—conserved in gene content (typically 13 protein-coding genes, 22 tRNAs, and 2 rRNAs) and readily obtainable—are widely used in Coleoptera for phylogeny, species identification, and population genetics (Cameron 2014b). However, for *Lucidina*, and particularly *L. vitalisi*, the published literature and databases still lack a fully annotated mitogenome. Therefore, we assembled and annotated the complete mitochondrial genome of *L. vitalisi* from whole-genome sequencing data. The mitochondrial genome was extracted by aligning the sequencing reads to the reference mitogenome of *Abscondita anceyi* (GenBank MH020192)*, Diaphanes citrinus* (GenBank MH651351)*, Photinus pyralis* (GenBank KY778696). Using genome structural features and sequence signals, we evaluated its phylogenetic placement within the Lampyrinae subfamily, thereby providing a key resource for comparative genomics and conservation genetics of East Asian “dark” fireflies.

## Materials and methods

**Figure 1.**
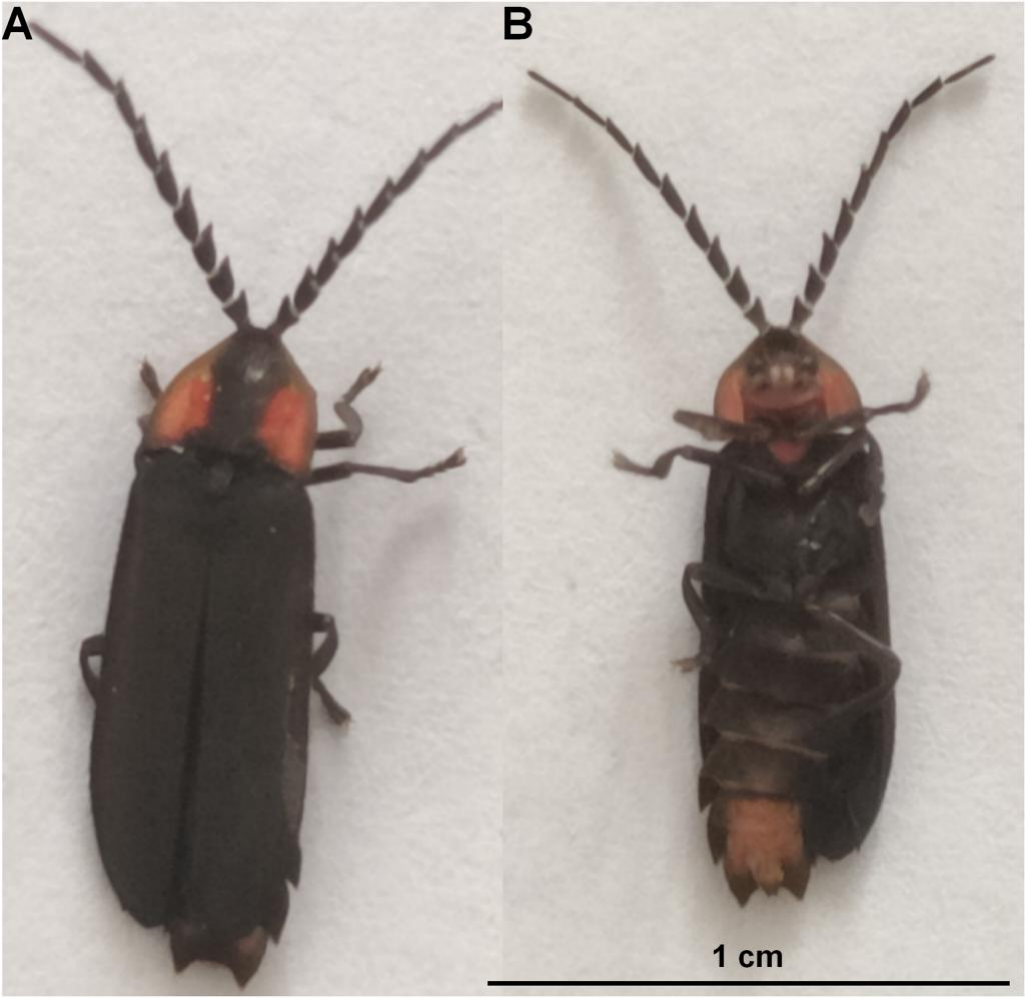
Reference image of *Lucidina vitalisi*. (A) Dorsal view. (B) Ventral view. Both photographs were taken by the author of this article, Xiao-Hua Guo.

In May 2023, live specimens of *L. vitalisi* were collected from Baishanzu National Park in Qingyuan County, Lishui City, Zhejiang Province, China (27°45′39.60″N, 119°11′52.80″E). Specimens were captured at night using a handheld insect net. The species were confirmed through the descriptions by Keller and Martin (2024) and Jeng (2008). Observations of key characteristics revealed that males had noticeably serrated antennae, a red pronotum with a central dark stripe, uniformly dark elytra, a tiny punctiform abdominal light organ, and slight sexual dimorphism in size (males ranging from approximately 8.7–10.2 mm). After being photographed using a Nikon D850, the specimens were briefly chilled to aid handling. Small samples of thoracic flight muscle were then dissected from the individuals and preserved in 95% ethanol. The euthanized specimens were also stored in 95% ethanol. These specimens are now housed in the zoological specimen room of the College of Ecology at Lishui University, under voucher number LSU-ZJ2023-05-121 (Figure 1). For further information, Dr. Xiao-Li Fan (Fanlilly@163.com) can be contacted.

Genomic DNA was subsequently extracted from the preserved muscle tissue using the Rapid Animal Genomic DNA Isolation Kit (Sangon, Shanghai, China), and the resulting DNA was used for whole-genome sequencing. Libraries with a 350-bp insert size were prepared using the TruSeq Nano^™^ kit (Illumina, San Diego, CA), and the whole genome was sequenced using the Illumina HiSeq 2500 platform, generating 150-bp paired-end reads. A total of 4.92 Gb of raw data was generated, of which 4.83 Gb of clean data was retained after removing low-quality reads and adapter sequences with Fastp v0.20.0 (Chen et al. 2018). After quality filtering, the reads were aligned to the reference mitogenome of *Abscondita anceyi* (GenBank MH020192)*, Diaphanes citrinus* (GenBank MH651351)*, Photinus pyralis* (GenBank KY778696) using BWA-MEM v0.7.17 (Li 2013). From this alignment, the mitochondrial genome was assembled into a circular draft contig using SPAdes v4.10 (Prjibelski et al. 2020). To eliminate any reference bias, *de novo* assembly was also performed with GetOrganelle v1.7.7 (Jin et al. 2020) (k-mers 21–127). The resulting contig matched the SPAdes draft and included all 37 standard mitochondrial genes and one non-coding region. This 14,882 bp contig was finalized as the mitogenome, circularized, and trimmed using MitoZ v2.4 (Meng et al. 2019), and polished twice with Pilon v1.24 (Walker et al. 2014). Genome annotation was carried out using the stand-alone MITOS2 tool (Donath et al. 2019) and cross-verified with NCBI BLAST+ v2.28 (Camacho et al. 2009), GeneWise (Birney et al. 2004), MiTFi (Jühling et al. 2012), and Infernal v1.1 (Nawrocki and Eddy 2013). Manual curation was performed in Geneious Prime v.2024.0.7 (Geneious 2025). A circular genome map was generated using Proksee (Grant et al. 2023). Coverage depth was verified by realigning raw reads to the final assembly using Bowtie2 v2.3.4 (Langmead and Salzberg 2012), with per-base depth calculated using SAMtools v1.16.1 (Li et al. 2009) and visualized with ggplot2 (Wickham 2016; Figure S1).

We selected mitochondrial genome sequences from 12 species within the Lampyridae family for this phylogenetic analysis. Although more mitochondrial genomes are available, these 12 species were chosen to capture the diversity of the family and to provide a robust basis for our analysis. In addition, the mitochondrial genome of *L. vitalisi*, newly sequenced in this study, was included. *Rhagophthalmus ohbai* (Coleoptera: Rhagophthalmidae) was selected as the outgroup for our phylogenetic reconstructions. The 13 protein-coding genes (*ND1*, *ND2*, *COX1*, *COX2*, *COX3*, *ATP6*, *ATP8*, *ND3*, *ND4*, *ND4L*, *ND5*, *ND6*, *CYTB*) were systematically extracted using PhyloSuite v1.2.1 (Zhang et al. 2020), and subsequent multiple sequence alignment was carried out with MAFFT v7.388 (Katoh and Standley 2013) under default parameters, where gaps were treated as “missing data” and were not imputed. Phylogenetic relationships were inferred using maximum likelihood analysis as implemented in IQ-TREE v2.1.2 (Minh et al. 2020), with the best-fit substitution model (GTR+F + I + G4) determined through ModelFinder (Kalyaanamoorthy et al. 2017). Branch support was evaluated with 1,000 bootstrap replicates. The resulting phylogenetic tree was visualized and annotated using the Interactive Tree of Life (ITOL v6) (Letunic and Bork 2021).

## Results

**Figure 2.**
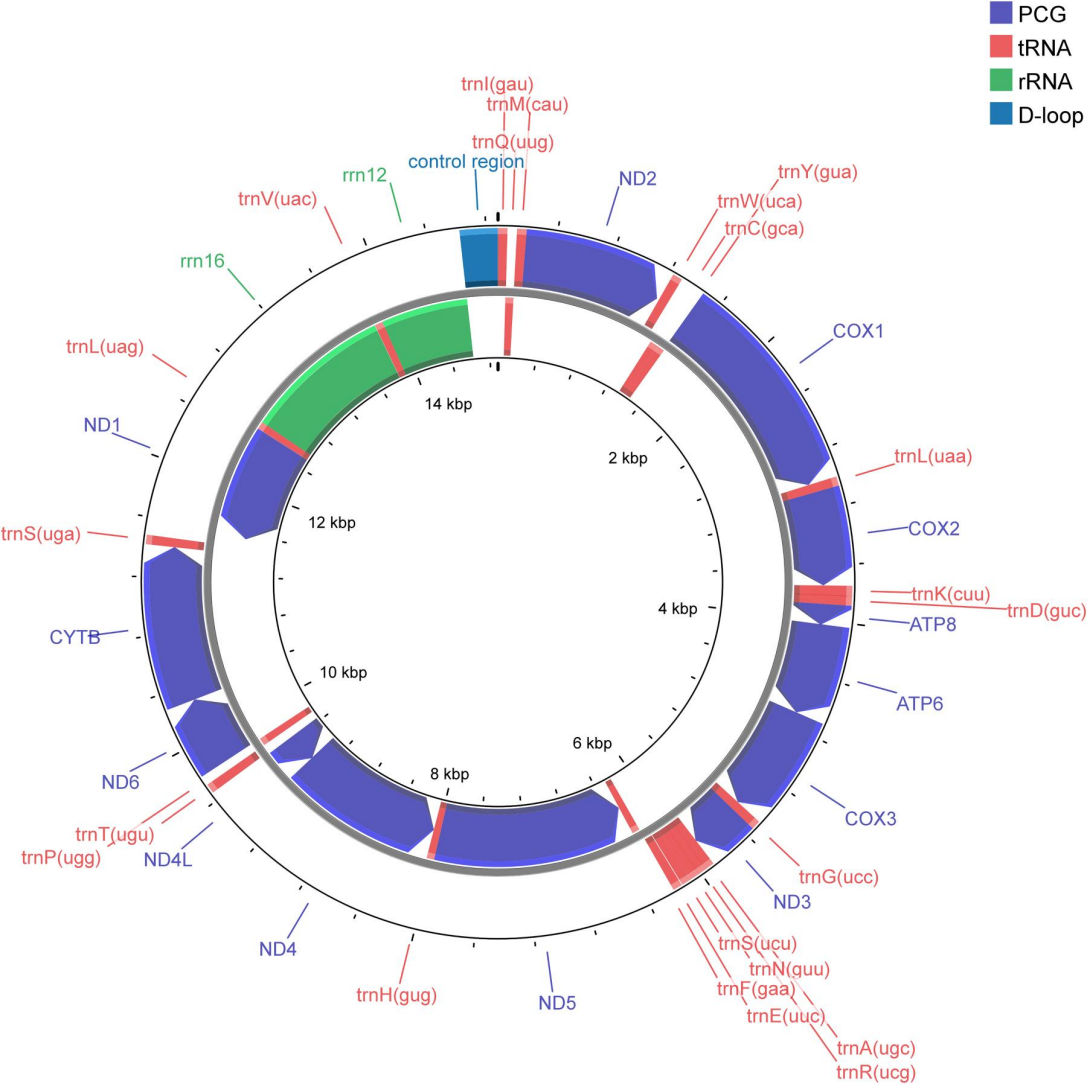
Circular map of the *Lucidina vitalisi* mitochondrial genome. Arrows indicate transcriptional directions.

The complete mitochondrial genome of *L. vitalisi* is 14,882 bp in length and contains a typical set of 37 genes: 13 protein-coding genes, 22 tRNAs, two rRNAs, and one control region (D-loop). The overall nucleotide composition is biased toward AT, with *A* = 41.28%, *T* = 36.87%, *G* = 8.63%, and *C* = 13.22%, resulting in a low G + C content of 21.85%. Twenty-three genes (9 PCGs and 14 tRNAs) are encoded on the majority strand, while the remaining 14 genes (4 PCGs, 8 tRNAs, and 2 rRNAs) are located on the minority strand. All PCGs initiate with a standard ATN start codon, except for *nd1*, and most terminate with TAA or TAG; however, COX*1*, *COX2*, *COX3*, and *ND5* end with an incomplete T stop codon. The tRNA genes vary in size from 60 to 70 bp. The lengths of the large (*rrn16*) and small (*rrn12*) ribosomal RNA genes are 1248 bp and 731 bp, with G + C contents of 18.43% and 20.38%, respectively. The control region (D-loop), situated between *rrn12* and *trnI*, is 259 bp long and exhibits a G + C content of 10.04% (Figure 2). We report per-gene lengths and A/T contents in Table S1.

**Figure 3.**
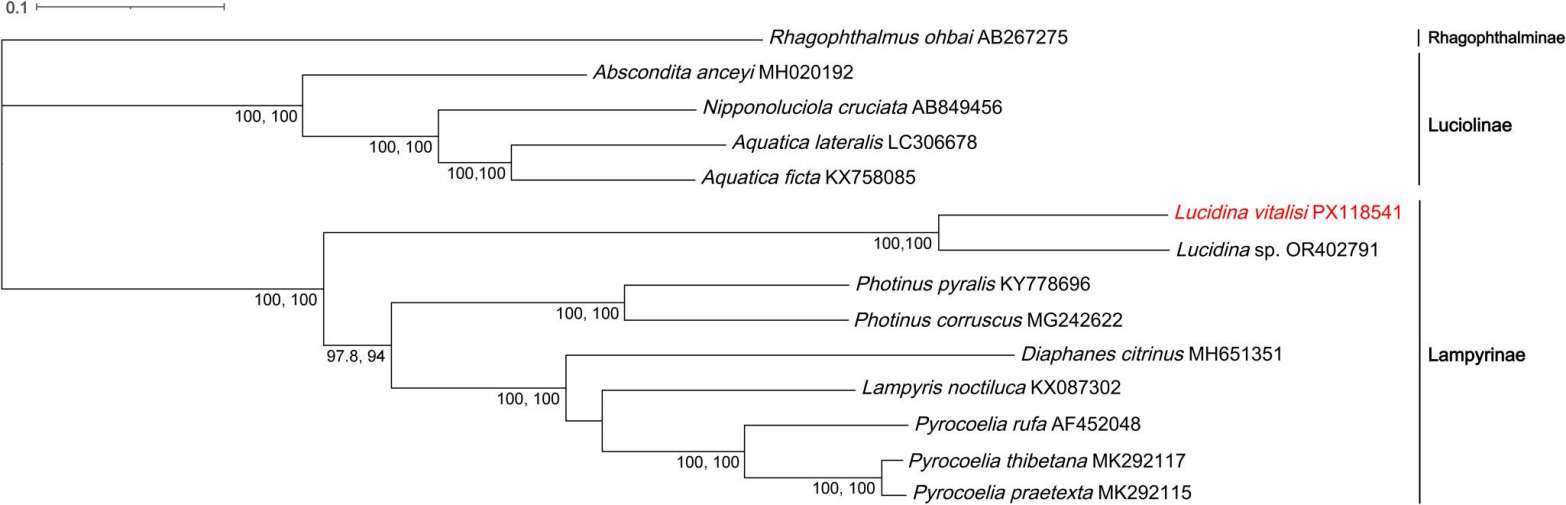
Maximum-likelihood phylogenetic tree based on the whole mitogenomes of *Lucidina vitalisi* and 12 species of Lampyridae family. Bootstrap values >70% are displayed above the branches. These values represent the SH-aLRT and UFBoot support for each corresponding node in the tree. Subfamily names for the groups are indicated in the tree, including Lampyrinae and Luciolinae for species in the Lampyridae family, and Rhagophthalminae for species in the Rhagophthalmidae family. Tribe information for all species used in this study is provided in Table S2. The following sequences were used: *Lucidina vitalisi* (PX118541; this study), *Lucidina* sp. (OR402791; unpublished), *Pyrocoelia praetexta* (MK292115; Chen et al. 2019), *Pyrocoelia thibetana* (MK292117; Chen et al. 2019), *Pyrocoelia rufa* (AF452048; Bae et al. 2004), *Lampyris noctiluca* (KX087302; unpublished), *Diaphanes citrinus* (MH651351; Yang and Fu 2019), *Photinus corruscus* (MG242622; unpublished), *Photinus pyralis* (KY778696; Fallon et al. 2018), *Aquatica ficta* (KX758085; Wang et al. 2017), *Aquatica lateralis* (LC306678; unpublished), *Nipponoluciola cruciata* (AB849456; unpublished), *Abscondita anceyi* (MH020192; Hu and Fu 2018), and *Rhagophthalmus ohbai* (AB267275; Li et al. 2007).

The ML tree from 13 mitochondrial PCGs places *L. vitalisi* as sister to *Lucidina* sp. with maximal support, firmly within Lampyrinae and clearly separated from Luciolinae (*Abscondita*, *Nipponoluciola*, *Aquatica). Photinus* and *Pyrocoelia* are each recovered as monophyletic with full support; most other backbone nodes have ≥90% support. The tree is rooted with *Rhagophthalmus ohbai* (Figure 3).

## Discussion and conclusion

The newly assembled and annotated mitogenome of *L. vitalisi* adds an East Asian, day-active (“dark”) lampyrid to the growing set of firefly mitochondrial resources and fills a conspicuous gap for *Lucidina* spp. at the whole-mitogenome level. As in other beetles, the genome comprises the canonical 37 genes and retains the ancestral insect gene order, including the conserved IQM tRNA cluster (*trnI*, *trnQ*, *trnM*) and rRNA–control-region arrangement, consistent with broad insect patterns reported for mitogenomes and for Coleoptera specifically (Cameron 2014a; Saito et al. 2025). The overall strong A + T bias also aligns with values typical of Lampyridae; for instance, *Nipponoluciola cruciata* (GenBank AB849456) displays approximately 76–77% A + T in its mitochondrial DNA (Maeda et al. 2017), and similar A + T content is observed in other Lampyridae species, such as *Pyrocoelia rufa* (∼77.4%, Bae et al. 2004) and *Diaphanes citrinus* (∼78.18%, Yang and Fu 2019).

Our phylogenetic analysis, based on 13 concatenated PCGs, identifies *L. vitalisi* as the sister species to an undescribed species of *Lucidina*, both belonging to the Lucidotini tribe and nested within the subfamily Lampyrinae. *L. vitalisi* and *Lucidina* sp. cluster together with other species, including *Photinus*, *Pyrocoelia*, *Lampyris*, and *Diaphanes*, all of which exhibit strong phylogenetic support. These relationships are consistent with the higher-level phylogeny of Lampyrinae, as reevaluated in Martin et al. (2019). The position of *Lucidina* within Lampyrinae, showing affinities near lucidotine lineages as per recent revisions, aligns with ongoing taxonomic updates that have refined tribe concepts and clarified genus boundaries across lampyrines (Ballantyne et al. 2022; Keller and Martin 2024). Consequently, our mitogenome provides an independent line of evidence supporting these recent reclassifications and offers an anchor for future phylogenomic integration.

We report the complete mitogenome of the day-active firefly *L. vitalisi*, which shows the canonical 37-gene architecture, strong A + T bias, and a notably short control region (259 bp) with otherwise typical lampyrid features. Phylogenetic analyses of 13 PCGs place *L. vitalisi* as sister to *Lucidina* sp. with maximal support, firmly within Lampyrinae and distinct from Luciolinae. This genome fills a taxonomic gap and provides a useful reference for comparative, population, and conservation genetics.

## Supplementary Material

Table S1.docx

Figure S1.doc

Table S2.docx

## Data Availability

The genome sequence data supporting this study are openly available in GenBank of NCBI at https://www.ncbi.nlm.nih.gov under the accession number PX118541. The associated BioProject, SRA, and Biosample numbers are PRJNA1303814, SRR34931685, and SAMN50536467, respectively.
