# Supplementary material for: The complete mitochondrial genome of Lucidina vitalisi (Coleoptera: Lampyridae) and its phylogenetic analysis
Source: Mitochondrial DNA B Resour. 2025 Nov 19;10(12):1205–9. doi: 10.1080/23802359.2025.2590334 (PMC12636536; doi:10.1080/23802359.2025.2590334)
Supplement: Table S1.docx [file TMDN_A_2590334_SM4910.docx]

**Table S1**. *Lucidina vitalisi* gene lengths and A/T percentage

| **Gene Name** | **Type** | **Start** | **End** | **Strand** | **Length (bp)** | **AT (%)** |
| --- | --- | --- | --- | --- | --- | --- |
| *ND2* | PCG | 194 | 1210 | + | 1017 | 80.33 |
| *COX1* | PCG | 1464 | 3003 | + | 1540 | 70.84 |
| *COX2* | PCG | 3067 | 3745 | + | 679 | 74.52 |
| *ATP8* | PCG | 3880 | 4032 | + | 153 | 83.66 |
| *ATP6* | PCG | 4029 | 4697 | + | 669 | 77.58 |
| *COX3* | PCG | 4697 | 5480 | + | 784 | 74.36 |
| *ND3* | PCG | 5543 | 5896 | + | 354 | 77.68 |
| *ND5* | PCG | 6280 | 7978 | - | 1699 | 79.11 |
| *ND4* | PCG | 8042 | 9370 | - | 1329 | 79.61 |
| *ND4L* | PCG | 9367 | 9639 | - | 273 | 78.39 |
| *ND6* | PCG | 9786 | 10289 | + | 504 | 82.14 |
| *CYTB* | PCG | 10289 | 11419 | + | 1131 | 73.65 |
| *ND1* | PCG | 11561 | 12511 | - | 951 | 77.92 |
| *rrn16* | rRNA | 12575 | 13822 | - | 1248 | 81.57 |
| *rrn12* | rRNA | 13894 | 14624 | - | 731 | 79.62 |
| *trnI* | tRNA | 1 | 64 | + | 64 | 76.56 |
| *trnQ* | tRNA | 62 | 129 | - | 68 | 86.76 |
| *trnM* | tRNA | 129 | 193 | + | 65 | 76.92 |
| *trnW* | tRNA | 1227 | 1293 | + | 67 | 79.1 |
| *trnC* | tRNA | 1340 | 1409 | - | 70 | 80 |
| *trnY* | tRNA | 1410 | 1471 | - | 62 | 79.03 |
| *trnL* | tRNA | 3004 | 3066 | + | 63 | 69.84 |
| *trnK* | tRNA | 3746 | 3815 | + | 70 | 72.86 |
| *trnD* | tRNA | 3814 | 3879 | + | 66 | 90.91 |
| *trnG* | tRNA | 5481 | 5542 | + | 62 | 88.71 |
| *trnA* | tRNA | 5895 | 5956 | + | 62 | 85.48 |
| *trnR* | tRNA | 5956 | 6015 | + | 60 | 70 |
| *trnN* | tRNA | 6015 | 6078 | + | 64 | 81.25 |
| *trnS* | tRNA | 6079 | 6146 | + | 68 | 79.41 |
| *trnE* | tRNA | 6152 | 6214 | + | 63 | 90.48 |
| *trnF* | tRNA | 6213 | 6276 | - | 64 | 82.81 |
| *trnH* | tRNA | 7979 | 8042 | - | 64 | 79.69 |
| *trnT* | tRNA | 9656 | 9720 | + | 65 | 84.62 |
| *trnP* | tRNA | 9721 | 9784 | - | 64 | 78.12 |
| *trnS* | tRNA | 11418 | 11481 | + | 64 | 78.12 |
| *trnL* | tRNA | 12513 | 12574 | - | 62 | 75.81 |
| *trnV* | tRNA | 13825 | 13893 | - | 69 | 85.51 |
| Control region | Control region | 14625 | 1 | / | 259 | 89.96 |
