# Supplementary material for: The complete mitochondrial genome of Lucidina vitalisi (Coleoptera: Lampyridae) and its phylogenetic analysis
Source: Mitochondrial DNA B Resour. 2025 Nov 19;10(12):1205–9. doi: 10.1080/23802359.2025.2590334 (PMC12636536; doi:10.1080/23802359.2025.2590334)
Supplement: Figure S1.doc [file TMDN_A_2590334_SM4909.doc]

**The first complete mitochondrial genome of *Lucidina vitalisi* (Coleoptera: Lampyridae) and its phylogenetic analysis**

Xiao-Hua Guo, Xiao-Li Fan, Zi-Long Zhong, Yan-Yun Xiong, Su-Mei Wu, Jin-Yang Li, You-Jun Wu

**Figure S1.** Depth of coverage for *Lucidina vitalisi* mitochondrial genome. X and Y axis present nucleotide position of *L. vitalisi* mitochondrial genome and coverage depth, respectively.
