# Supplementary material for: The complete mitochondrial genome of Lucidina vitalisi (Coleoptera: Lampyridae) and its phylogenetic analysis
Source: Mitochondrial DNA B Resour. 2025 Nov 19;10(12):1205–9. doi: 10.1080/23802359.2025.2590334 (PMC12636536; doi:10.1080/23802359.2025.2590334)
Supplement: Table S2.docx [file TMDN_A_2590334_SM4908.docx]

**Table S2**. Tribe classification for species of Lampyridae and Rhagophthalmidae

| **Species** | **Family** | **Subfamily** | **Tribe** |
| --- | --- | --- | --- |
| *Lucidina vitalisi* | Lampyridae | Lampyrinae | Lucidotini |
| *Lucidina* sp. | Lampyridae | Lampyrinae | Lucidotini |
| *Pyrocoelia praetexta* | Lampyridae | Lampyrinae | Lucidotini |
| *Pyrocoelia thibetana* | Lampyridae | Lampyrinae | Lucidotini |
| *Pyrocoelia rufa* | Lampyridae | Lampyrinae | Lucidotini |
| *Lampyris noctiluca* | Lampyridae | Lampyrinae | Lampyrini |
| *Diaphanes citrinus* | Lampyridae | Lampyrinae | Lampyrini |
| *Photinus corruscus* | Lampyridae | Lampyrinae | Photinini |
| *Photinus pyralis* | Lampyridae | Lampyrinae | Photinini |
| *Aquatica ficta* | Lampyridae | Luciolinae | Luciolini |
| *Aquatica lateralis* | Lampyridae | Luciolinae | Luciolini |
| *Nipponoluciola cruciata* | Lampyridae | Luciolinae | Luciolini |
| *Abscondita anceyi* | Lampyridae | Luciolinae | Absconditini |
| *Rhagophthalmus ohbai* | Rhagophthalmidae | Rhagophthalminae | Rhagophthalmini |
